# Identification of Actionable Gene Variants in Pulmonary Large-Cell Neuroendocrine Carcinoma: A Real-World Analysis of a Polish Cohort

**DOI:** 10.3390/ijms27072939

**Published:** 2026-03-24

**Authors:** Adam Szpechcinski, Magdalena Pelc, Urszula Lechowicz, Malgorzata Szolkowska, Joanna Moes-Sosnowska, Piotr Rudzinski, Emil Wojda, Paulina Skronska, Elzbieta Podgorska, Krystyna Maszkowska-Kopij, Mateusz Polaczek, Tadeusz Orlowski, Renata Langfort, Joanna Chorostowska-Wynimko

**Affiliations:** 1 Department of Genetics and Clinical Immunology, The Institute of Tuberculosis and Lung Diseases, 01-138 Warsaw, Poland; u.lechowicz@igichp.edu.pl (U.L.); j.moes@igichp.edu.pl (J.M.-S.); jaguspaulina@gmail.com (P.S.); ep@it-badger.com (E.P.); j.chorostowska@igichp.edu.pl (J.C.-W.); 2 Department of Pathology, The Institute of Tuberculosis and Lung Diseases, 01-138 Warsaw, Poland; m.szolkowska@igichp.edu.pl (M.S.); r.langfort@igichp.edu.pl (R.L.); 3 Clinical Department of Thoracic Surgery, The Institute of Tuberculosis and Lung Diseases, 01-138 Warsaw, Poland; p.rudzinski@igichp.edu.pl (P.R.); t.orlowski@igichp.edu.pl (T.O.); 4 Clinical Department of Thoracic Tumors, The Institute of Tuberculosis and Lung Diseases, 01-138 Warsaw, Poland; emilwojda@wp.pl (E.W.); m.polaczek@igichp.edu.pl (M.P.); 5 Outpatient Clinic, The Institute of Tuberculosis and Lung Diseases, 01-138 Warsaw, Poland; krystynakopij@gmail.com

**Keywords:** pulmonary large-cell neuroendocrine carcinoma (LCNEC), next-generation sequencing (NGS), single nucleotide variant (SNV), gene fusion variant, targeted therapy

## Abstract

Pulmonary large-cell neuroendocrine carcinoma (LCNEC) is a rare lung malignancy characterized by an aggressive clinical course and an unfavorable prognosis. Next-generation sequencing (NGS) has revealed that LCNECs exhibit molecular features resembling either small-cell lung carcinoma (SCLC-like LCNEC) or non-small cell lung carcinoma (NSCLC-like LCNEC). This study aimed to characterize the incidence of actionable gene variants in a retrospective cohort of LCNEC patients using a targeted NGS approach. Microscopic diagnosis was established according to the 2021 World Health Organization (WHO) classification using a standard immunohistochemical (IHC) panel. In total, 216 LCNEC tumor samples were analyzed for molecular variants in 17 genes using the RNA-based Archer FusionPlex Lung NGS assay (Integrated DNA Technologies, USA) and the MiSeq platform (Illumina, USA)—an algorithm utilized for routine NSCLC diagnosis. Overall, 46 variants were identified in 46/216 (21.3%) tumor samples, with 28/216 (13%) LCNECs harboring at least one actionable molecular variant potentially targetable by registered or investigational agents. *KRAS* variants (5%; including *G12C* at 2%) and *PIK3CA* variants (5%) were the most prevalent, followed by *RET* single-nucleotide variants (3%), uncommon *EGFR* variants (1%), and *BRAF* class II and III variants (<1%). Notably, no classical *EGFR* exon 18–21 mutations nor *ALK*, *FGFR1/2/3*, or *ROS1* alterations (mutations or fusions) were detected, despite the technical capability of the assay to identify such variants. A novel in-frame gene fusion (*TMEM79::NTRK1*) was identified in a single tumor sample (0.5%). Our results confirm that LCNECs harbor potentially targetable alterations in *KRAS*, *PIK3CA*, *RET*, *BRAF*, and *NTRK1*, albeit at lower frequencies than those typically observed in NSCLC.

## 1. Introduction

Lung neuroendocrine neoplasms account for approximately 20% of all lung malignancies and represent a heterogeneous group of carcinomas originating from specialized, peptide- and amine-producing neuroendocrine cells [1]. Pulmonary large-cell neuroendocrine carcinoma (LCNEC) is a highly aggressive tumor characterized by neuroendocrine differentiation, representing approximately 3% of all resected carcinomas [2]. The prognosis for pulmonary LCNEC is generally poor; the median overall survival often does not exceed one year, with five-year survival rates of only 8% for stage III and 0% for stage IV disease [3].

According to the 2021 World Health Organization (WHO) classification, lung neuroendocrine neoplasms are divided into neuroendocrine tumors (typical and atypical carcinoids) and neuroendocrine carcinomas, which include LCNEC and small-cell lung carcinoma (SCLC) [4]. LCNEC containing any non-neuroendocrine, non-small cell lung carcinoma (NSCLC) component is classified as combined LCNEC. Diagnosis requires the identification of neuroendocrine morphology and the expression of at least one neuroendocrine marker (e.g., chromogranin A, synaptophysin, CD56, or INSM1) via immunohistochemical (IHC) testing [2]. To date, no standard treatment regimen has been established, and the optimal management of LCNEC remains under debate [5]. For early-stage LCNEC, surgery is the cornerstone of treatment. However, while improved outcomes following surgery have been demonstrated, data regarding the efficacy of adjuvant chemotherapy in stage I disease remain inconclusive [6]. For unresectable LCNEC, a multimodal approach comprising systemic chemotherapy and radiotherapy is recommended [7]. Nevertheless, 40–50% of patients are diagnosed with metastatic disease at the time of presentation [2]. Due to the low overall incidence of LCNEC and the clinical and molecular characteristics it shares with both SCLC and NSCLC, first-line treatment for metastatic disease is not yet standardized. Most available data on chemotherapy effectiveness stem from retrospective analyses of small patient cohorts receiving regimens typically used for SCLC (platinum-etoposide) or NSCLC (platinum–taxane, platinum–irinotecan, or platinum–pemetrexed), alongside a few prospective clinical trials [8,9,10,11,12]. Data regarding the efficacy of immunotherapy in LCNEC are encouraging but limited, derived primarily from case reports, case series, and real-world evidence. Several clinical trials evaluating combination strategies with anti-PD-L1 therapies in LCNEC are currently ongoing [13,14].

Targetable driver alterations are uncommon in LCNEC but occur more often in combined LCNEC with an adenocarcinoma component [15]. Several studies reported on classical NSCLC-driver alterations, though with varying frequencies, in anaplastic lymphoma receptor tyrosine kinase *(ALK*), B-raf proto-oncogene, serine/threonine kinase (*BRAF*), epidermal growth factor receptor (*EGFR*), erb-B2 receptor tyrosine kinase 2 (*ERBB2*), KRAS proto-oncogene, GTPase (*KRAS*), MET proto-oncogene, receptor tyrosine kinase (*MET*), neurotrophic receptor tyrosine kinase 1/2/3 (*NTRK1/2/3*), phosphatidylinositol-4,5-bisphosphate 3-kinase catalytic subunit alpha (*PIK3CA*), rearranged during transfection proto-oncogene (*RET*), ROS proto-oncogene 1, receptor tyrosine kinase (*ROS1*) genes found in LCNEC tumors using next-generation sequencing (NGS) techniques [16,17,18,19]. Clinical experience with targeted agents in LCNEC remains primarily limited to case reports. These have demonstrated promising outcomes in patients with *ALK* [20,21,22,23], *BRAF* [24,25], *EGFR* [26,27], and *RET* [28,29] alterations, establishing a strong rationale for larger prospective clinical trials in this patient population. In this study, we evaluated more than 200 pulmonary LCNECs for actionable variants in genes commonly used as predictive biomarkers for NSCLC targeted therapies, utilizing a well-established targeted NGS assay. The objective was to assess the frequency of clinically significant alterations in a Polish cohort of LCNEC patients and to estimate the number of subjects who could potentially benefit from personalized treatment if they were to undergo routine molecular testing as part of standard diagnostic procedures. Currently, molecular testing is a critical consideration in LCNEC management given the recent development of effective targeted agents; however, neither dedicated recommendations nor formal guidelines have been established to date [30].

## 2. Results

### 2.1. Technical Performance of NGS Assay

NGS provided valid raw genomic data for all samples analyzed. All NGS reactions were conducted with high-quality parameters: QC30 range of 85.0–94.0% (quality norm according to manufacturer—QC > 85%), cluster density spreading 1101–1564 k/mm^2^ (optimum according to manufacturer 1200–1400 k/mm^2^). The median (min–max) total number of fragments (read pairs) that were present in the original FASTQ file was 1,287,721 (1,000,487–1,969,612) at the recommended minimum of 1 mln reads, and the median (min–max) percentage of on-target reads was 98.3% (93.9–99.1%). A QC metric (the average number of unique control molecules that were characterized as RNA fragments) of at least 10 was required to support the targets of the assay. The median QC metric of analyzed samples was 213.5 (range: 37.4–416.8).

### 2.2. The Detection of Molecular Variants in Clinical Samples by NGS Assay

The pulmonary LCNEC tumor samples were tested for single nucleotide variants (SNVs), deletion/insertion (DELINS), splicing variants, exon-skipping variants, and gene fusion variants by targeted NGS using the FusionPlex Lung panel (Integrated Sciences, Chatswood, CA, USA) in specific genomic regions (including 323 amplicons from 17 genes associated with lung cancer; Tables S1 and S2). Only non-synonymous, in-frame variants in the coding regions of targeted genes that potentially modulate protein structure and function were analyzed.

**Figure 1:**
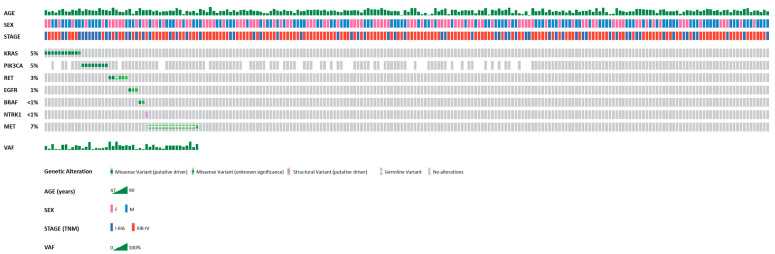
The OncoPrint showing the distribution and variant allele frequency (VAF) of molecular alterations within 17 targeted genes, detected in 216 pulmonary large-cell neuroendocrine carcinoma (LCNEC) samples by targeted NGS assay. Additionally, basic demographic (age, sex) and pathological (clinical stage) data of patients with LCNEC are presented. The variant type is labeled in the color legend and tumor samples in columns. The samples not evaluated for *PIK3CA* variants are shown as blank boxes.

In this study, we identified 21 unique single-nucleotide variants within the 6 genes in 45/216 (20.8%) tumor samples and a fusion variant within a single sample (0.5%; Table 1, Figure 1). In total, 46 variants have been found in 46/216 (21.3%) tumor samples (Table S3). No sample presented more than one variant in the analysis. Twenty-six out of 45 (58%) samples presented an SNV known to be oncogenic/likely oncogenic with mostly gain-of-function effect on the protein. The remaining 19/45 (42%) samples presented an SNV classified as benign/likely benign or a variant of unknown clinical significance.

Sixteen missense or DELINS variants in *BRAF* (p.Gly469Ala, p.Asn581Lys), *EGFR* (p.Glu330Lys, p.Gly598Arg), *KRAS* (p.Gly12Cys, p.Gly12Val, p.Gly12Leu, p.Ala11_Gly12 delinsProVal), *PIK3CA* (p.Glu542Lys, p.Glu545Lys, p.Gln546Lys, p.His1047Leu, p.His1047Arg), and *RET* (p.Cys609Gly, p.Ser649Leu, p.Met918Thr) genes were frequently reported in databases (listed in Section 4.6) as somatic in various solid tumor types. We also identified several missense variants in *MET* (p.Asn375Ser) and *RET* (p.Gly691Ser, p.Tyr791Phe) classified as benign/likely benign germline polymorphisms in clinical databases. The classification of remaining variants detected in *EGFR* (p.Ala743Ser) and *MET* (p.Arg970Cys) genes is conflicting or unknown.

A single tumor sample (female, 72 years old, stage IVB) demonstrated an in-frame fusion variant formed by transmembrane protein 79 *(TMEM79*) and *NTRK1* genes, both located on chromosome 1, with a breakpoint occurring in exon 3 of *TMEM79* (chr1:156256264) and exon 2 of *NTRK1* (chr1:156834146; Figure 2). This fusion variant has not been reported in databases nor reviewed in the scientific literature. The predicted fusion protein retains the entire tyrosine kinase domain with conserved catalytic motifs (Figure 3).

No significant associations have been found between genetic variants and demographic (sex, age) or clinical parameters (tumor stage).

Furthermore, no *ALK*, *FGFR1*, *FGFR2*, *FGFR3*, *ROS1* alterations (mutations, fusions) were found in our pulmonary LCNEC cohort despite technical capability of our NGS assay to detect such variants.

## 3. Discussion

In this study, we evaluated pulmonary LCNECs for molecular alterations commonly found in NSCLC and other solid tumors that carry predictive or prognostic value for targeted therapies and immunotherapies. Specifically, we utilized targeted NGS to identify actionable single-nucleotide variants (SNVs) and fusions.

To date, comprehensive genomic profiling studies revealed a C:G>A:T transversion rate of 38.7–47.5%, indicative of tobacco exposure, and high tumor mutational burden of 8.6–10.5 non-synonymous mutations per million base pairs in pulmonary LCNECs [31,32]. Furthermore, genomic and transcriptomic analyses have enabled the distinction of different molecular subtypes of LCNEC, which are large cell neuroendocrine carcinomas with molecular features similar to SCLC (SCLC-like LCNEC overlapping with LCNEC type II classification) and large cell neuroendocrine carcinomas with molecular features similar to NSCLC (NSCLC-like LCNECs overlapping with LCNEC type I classification) [16,31,32,33]. The SCLC-like LCNEC subtype is characterized by the inactivation of *RB1/TP53* genes, resulting in the lack of RB1 protein expression and frequent *RB1/TP53* co-mutations. It also features other alterations typical of small-cell lung carcinoma, such as *MYCL* amplification, while notably lacking *STK11* or *KRAS* mutations. This subtype typically exhibits low expression of neuroendocrine markers, specifically *ASCL1* (Achaete-Scute family BHLH transcription factor 1) and *DLL3* (delta-like canonical notch ligand 3), alongside the activation of Notch signaling pathway genes (*ASCL^low^/DLL3^low^/NOTCH^high^*) [2,13,34]. Conversely, the NSCLC-like LCNEC subtype is characterized by the presence of wild-type RB1 protein and a lack of *RB1/TP53* co-mutations. Instead, it frequently harbors additional molecular alterations in *BRAF*, *ERBB2*, *CDKN2A*, *KRAS*, *STK11*, or *KEAP1* genes, which may co-occur with *TP53* mutations. This subtype shows high expression of *ASCL1* and *DLL3* but exhibits suppression of the Notch signaling pathway (*ASCL^high^/DLL3^high^/NOTCH^low^*). This molecular classification has significant prognostic and predictive implications for treatment efficacy, as Type I (with *TP53* and *STK11/KEAP1* alterations) and Type II LCNECs (with *TP53* and *RB1* alterations) demonstrate heterogeneous responses to chemotherapy and immunotherapy [2,34]. In the present study, we did not perform molecular subtyping into SCLC-like and NSCLC-like categories, as this procedure has neither been standardized nor recommended in current clinical guidelines.

In our study, molecular variants have been found in 46/216 (21.3%) tumor samples. Twenty-eight out of two-hundred sixteen (13%) LCNEC tumors showed at least one molecular alteration potentially targetable by registered or investigational agents (Table 1 and Table S3). Among these alterations, oncogenic *KRAS* and *PIK3CA* variants have shown the highest frequency in tested samples (5% and 5%, respectively). Similar frequencies of oncogenic alterations in *KRAS* (6–10%) and *PIK3CA* (3–6%) genes have been reported in other studies using NGS techniques for molecular profiling of pulmonary LCNEC tumors [16,18,35,36]. Importantly, 6 out of 11 (55%) *KRAS* variants detected in our LCNEC cohort were p.Gly12Cys substitutions that offer a unique opportunity for therapeutic targeting using small-molecule inhibitors, adagrasib and sotorasib, currently approved by the U.S. Food and Drug Administration (FDA) and the European Medicines Agency (EMA) in NSCLC [37]. The available, early data on clinical efficacy of these drugs in *KRAS* G12C-mutated pulmonary LCNECs, though limited to a few case reports, show favorable antitumor responses [38,39]. The other (non-G12C) *KRAS* variants observed in our study also show potential for therapeutic inhibition by experimental drugs currently at different levels of clinical development [40,41]. No clinical evidence on the efficacy of PI3K inhibition in pulmonary LCNECs, to the best of our knowledge, has been published to date. Still, all *PIK3CA* gene alterations detected in our study are targetable by drugs that are already approved with indications to other solid tumors (e.g., alpelisib, copanlisib) or by novel investigational medicinal products (e.g., zovegalisib) [42].

Despite a large number of pulmonary LCNEC tumors analyzed in our study, we observed either no classical *EGFR* mutations (exon 19 deletions, p.Leu858Arg) nor class I *BRAF* variants (p.Val600Asp/Glu/Lys/Arg) that have been sporadically reported by other groups [16,18,32]. Instead, we identified several uncommon *EGFR* variants located in the extracellular domain (p.Glu330Lys, p.Gly598Arg) and the kinase domain of the protein (p.Ala743Ser), whose oncogenicity and sensitivity to EGFR-TKIs have not been fully elucidated. Likewise, only class II (p.Gly469Ala) and III (p.Asn581Lys) *BRAF* variants were found in two different LCNEC tumors. Substitutions at these positions in the *BRAF* gene have been previously observed in pulmonary LCNEC at low frequencies [25]. In a patient with metastatic LCNEC of the lung with *BRAF* p.Gly469Arg variant, treated with trametinib and dabrafenib combination, a stable disease has been maintained for 15 months. Unlike V600 mutations, non-V600 *BRAF* alterations are not yet well-validated as therapeutic targets, and clinical evidence for effective targeted treatments is still limited [43,44].

The RET proto-oncogene plays a critical role in normal neuroendocrine development, encoding a receptor tyrosine kinase that regulates cell growth, survival, and differentiation, particularly within neural-crest-derived cells and the enteric nervous system [45]. Ligand-independent activation of RET typically arises from gain-of-function mutations or the formation of fusion proteins. These fusions combine the intracellular kinase domain of RET with an N-terminal domain from a partner protein capable of dimerization [46].

Once altered, RET becomes constitutively active, persistently stimulating downstream signaling cascades such as the ERK and PI3K/Akt pathways, which promote cellular proliferation, survival, and metastasis [47]. While neuroendocrine tumors often arise from hormone-secreting cells of neural crest origin (such as those in medullary thyroid carcinoma, where RET alterations are identified in approximately 50% of patients), these alterations are notably rare in small-cell lung cancer [48]. In contrast, *RET* gene fusions are found in 1–2% of all non-small cell lung carcinomas [49]. In our study, we found *RET* gene mutations in six (2.7%) patients with pulmonary LCNEC, whereas three of these variants have been confirmed to be activating and oncogenic (p.Cys609Gly, p.Ser649Leu, p.Met918Thr), and potentially druggable with selective RET inhibitors. Selpercatinib has been found to be effective at treating NSCLC and certain thyroid cancers caused by changes to the *RET* gene [50,51]. Recent case reports show the successful use of selpercatinib as a first-line treatment in a patient with advanced pulmonary LCNEC harboring a *RET* fusion gene [28,29]. To date, activating RET alterations, including both point mutations and fusions, have rarely been reported in pulmonary LCNEC, with most occurrences documented only in isolated case reports [16,35]. Our findings suggest that the true prevalence of these alterations in LCNEC may be underestimated, potentially due to factors such as limited study cohort sizes or the technical constraints of specific NGS assays.

**Figure 2:**
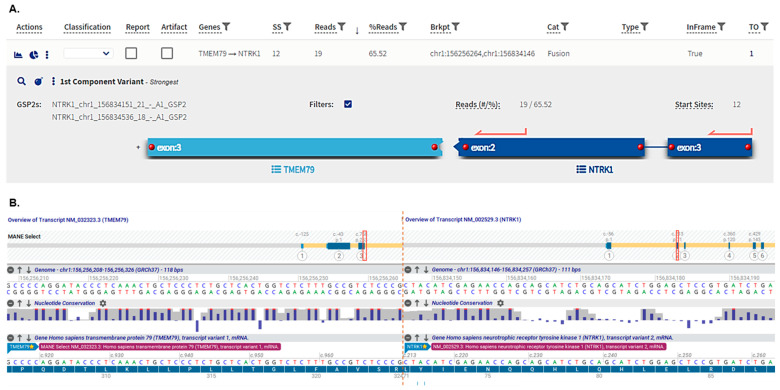
The graphic representation of *TMEM79::NTRK1* fusion, including exonic (TMEM79 NM_032323.3 exon 3; NTRK1 NM_002529.3 exon 2) and chromosomal (chr1:156256264, chr1:156834146) breakpoint positions based on the output data from the Archer Analysis software version 7.2 (ArcherDX, Inc., Boulder, CO, USA; (**A**)) and on the variant visualization by the Alamut Visual Plus v1.12 software (SOPHiA GENETICS, Inc., Boston, MA, USA; (**B**)).

**Figure 3:**
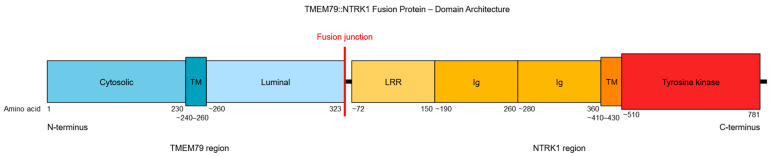
Predicted domain organization of the TMEM79::NTRK1 fusion protein. The fusion protein consists of the N-terminal portion of TMEM79 (aa 1–323) fused in-frame to the NTRK1 sequence starting from exon 2 (aa ~72–796). The predicted protein contains two transmembrane helices (TM) derived from TMEM79 and NTRK1, respectively. The extracellular portion includes leucine-rich repeat (LRR) and immunoglobulin-like domains (Ig) typical for TRKA. The cytoplasmic region retains the entire tyrosine kinase domain with conserved catalytic motifs. The fusion junction occurs between the luminal region of TMEM79 and the extracellular region of NTRK1.

In addition to SNV and DELINS variants, we detected a *TMEM79::NTRK1* fusion variant (t(1;1), NM_032323.3 exon A: 3; NM_002529.3 exon B: 2; (chr1:156256264,chr1:156834146)) in a single tumor sample. The resulting in-frame fusion protein comprises the N-terminal portion of TMEM79 fused to the NTRK1 sequence beginning at exon 2 (Figure 2). The predicted protein structure retains two transmembrane helices—derived from TMEM79 and NTRK1, respectively—while the extracellular portion includes the leucine-rich repeat and immunoglobulin-like domains characteristic of TRKA (Figure 3). Crucially, the cytoplasmic region preserves the entire tyrosine kinase domain, including all conserved catalytic motifs. The fusion junction is located between the luminal region of TMEM79 and the extracellular region of NTRK1. Notably, this fusion protein lacks a classical oligomerization domain (such as coiled-coil, zinc-finger, or WD domains) typically required for the ligand-independent activation of NTRK oncoproteins [52]. While over 80 NTRK fusion partners have been identified—most of which facilitate constitutive downstream signaling via introduced oligomerization domains—certain fusions, such as SCYL3::NTRK1, lack these motifs [53]. In such cases, the fusion partner may drive kinase self-activation through alternative mechanisms, such as inducing liquid–liquid phase separation (LLPS) [54]. Collectively, these structural features suggest that *TMEM79::NTRK1* likely encodes a functional kinase, and the fusion protein would likely remain sensitive to tropomyosin receptor kinase inhibitors targeting the ATP pocket of TRKA, including larotrectinib and entrectinib [55]. To our knowledge, this specific fusion variant has not been previously documented in the literature or genomic databases. However, other rare in-frame NTRK fusions, such as *RFWD2::NTRK1*, have been reported in pulmonary large-cell neuroendocrine carcinomas [32,56]. While members of the transmembrane (TMEM) protein family, including TMEM79, are recognized fusion partners in prostate carcinoma, they have not been previously described in lung cancer [57,58]. Compared to *ALK* and *RET* fusions, which are more prevalent in pulmonary LCNEC and typically associated with clinical sensitivity to tyrosine kinase inhibitors (TKIs), NTRK rearrangements appear to be sporadic molecular events in this malignancy [20,21,22,23,28,29,59,60,61,62].

Notably, 14 out of 216 (6.5%) LCNEC cases harbored the p.Asn375Ser (N375S) variant within the semaphorin domain of the MET protein—the ligand-binding region for hepatocyte growth factor (HGF). While this variant appears to be a common germline polymorphism [63], it has been shown to slightly reduce MET binding affinity for HGF [64]. More recently, research has demonstrated that N375S confers an exquisite binding affinity for HER2, enabling MET^N375S^ to interact with HER2 in a ligand-independent manner. This interaction drives aggressive squamous cell carcinomas of the head, neck, and lung and is associated with a poor prognosis [65]. Paradoxically, HER2 inhibitors—rather than c-MET inhibitors—have proven effective in restraining in vivo and in vitro models expressing MET^N375S^.

The MET p.Arg970Cys (R970C) mutation—also identified as R988C in alternative transcripts—is located in the juxtamembrane domain of the protein and was identified in a single LCNEC case in our study. Available data regarding the clinical significance of this variant remains conflicting. Some studies suggest that MET^R970C^ enhances oncogenic function, for instance, by promoting calpain-dependent generation of a p45 MET fragment that facilitates epithelial cell scattering and invasion in NSCLC [66] or by inducing constitutive MET phosphorylation that increases cell motility, migration, and colony formation in SCLC [67]. Conversely, other reports suggest that the p.R970C variant is non-oncogenic and has no significant effect on MET phosphorylation levels [68,69].

This study has several limitations. While the 17-gene NGS panel may appear limited in scope, it encompasses all clinically significant predictive biomarkers for FDA/EMA-approved targeted therapies in NSCLC, as established by the National Comprehensive Cancer Network (NCCN) [70] and the European Society for Medical Oncology (ESMO) guidelines [71]. Furthermore, the FusionPlex Lung panel is capable of detecting not only established actionable biomarkers but also emerging, sporadic, and novel rearrangements, including single-nucleotide variants (SNVs), insertions/deletions (indels), and gene fusions within the targeted genomic regions [72]. The *STK11* and *KEAP1* mutations, which have emerged as predictive biomarkers of immunotherapy resistance in *KRAS*-positive NSCLC, were not included in the molecular analysis since this study is focused on actionable variants related to targeted therapies [73]. Additionally, all NGS analyses were performed using total RNA isolated from LCNEC tissue specimens, rather than the more conventional approach of separate DNA and RNA isolation for the detection of SNVs, copy number variants (CNVs), and fusions, respectively. This focused method offers several technical advantages for clinical FFPE samples, e.g., higher RNA yield (the tissue is not subdivided for separate extractions, which is critical for small biopsies), and increased sensitivity (we achieved a high sequencing depth, at least 1 million reads per sample, allowing for variant detection down to 5% tumor cellularity). In our experience, this RNA-based targeted NGS approach has proven to be a reliable and informative diagnostic tool for the simultaneous detection of both known and novel actionable variants, providing pivotal data for therapeutic decision-making in NSCLC [74,75,76].

## 4. Materials and Methods

### 4.1. Patients

A total of 216 patients with pulmonary LCNEC were enrolled in this study (median age: 70 years; range: 47–90 years). According to the international Tumor-Node-Metastasis (TNM) staging system [77], 66 patients (31%) presented with operable localized or locally advanced disease (stages I–IIIA), while 150 patients (69%) had advanced metastatic disease (stages IIIB–IV). All patients were of Caucasian descent and were treatment-naïve prior to mutation testing. The study was approved by the Ethics Committee of the Institute of Tuberculosis and Lung Diseases in Warsaw, Poland (Reference: KB.0028.22.2025, 25 June 2025). Patient demographic and clinical characteristics are summarized in Table 2.

### 4.2. Pathological Diagnosis

The microscopic diagnosis of LCNEC has been established according to the 2021 World Health Organization (WHO) histological classification [4]. Microscopic criteria included morphology, the number of mitotic figures (>10/2 mm^2^), the presence of necrosis and distinct expression of at least one immunohistochemical neuroendocrine marker (chromogranin A, synaptophysin or CD56/NCAM). Ki-67 proliferation index was not a diagnostic criterion; however, a threshold >30% favored a diagnosis of carcinoma. Other immunohistochemical tests used included: pancytokeratin or cytokeratin 5/6, TTF-1 or napsin A and p40. The study did not analyze LCNEC and combined LCNEC separately, as it was usually impossible to identify both components in biopsies.

### 4.3. Types of Tumor Specimens

The tumor tissue specimens were obtained from patients with LCNEC during surgery or biopsy, following standard clinical procedures. In total, 216 FFPE tumor tissue samples were analyzed for molecular alterations. The median tumor cell content was 80% (range: 5–100%) in FFPE tissues. The minimum number of 1000 tumor cells required for NGS analysis was confirmed in every specimen.

### 4.4. RNA Extraction

FFPET blocks were cut into 5 μm thick sections and collected in sterile Eppendorf tubes. Total RNA was extracted from the FFPE tumor tissue sections using the spin column-based ReliaPrep FFPE Total RNA Miniprep System (Promega Corporation, Madison, WI, USA). The concentration and purity of RNA eluates were determined via spectrophotometry and fluorometry. RNA quality, specifically the integrity of amplifiable RNA, was evaluated using a qPCR-based assay (PreSeq RNA QC Assay, Integrated Sciences, Chatswood, CA, USA), according to the manufacturer’s protocols and established laboratory guidelines [78,79].

### 4.5. NGS Analysis of Gene Variants

Tumor RNA was sequenced and screened for SNVs and gene fusions using the FusionPlex Lung panel (Integrated Sciences, Chatswood, CA, USA). Initially, 44 (20%) samples were evaluated by the v1 panel, while the remaining 172 (80%) samples were analyzed using the extended v2 panel, which replaced the earlier version (Supplementary Tables S1 and S2). Briefly, RNA (250 ng) was reverse-transcribed to cDNA using random priming. cDNA quality was subsequently verified with the PreSeq RNA QC Assay (Integrated Sciences, Chatswood, CA, USA); only samples with a Cp < 29 proceeded to library construction according to the manufacturer’s protocol. Library concentration and quality were determined using the KAPA Universal Library Quantification Kit (Roche Diagnostics, Basel, Switzerland). Finally, libraries were normalized, multiplexed, and sequenced on the MiSeq platform using the MiSeq Reagent Kit, v3 (600 cycles) (Illumina, San Diego, CA, USA).

### 4.6. Bioinformatics and Computational Analysis of NGS Output Data

NGS data were analyzed using Archer Analysis software (v7.2; ArcherDX, Inc., Boulder, CO, USA) with the GRCh37 (hg19) reference genome. Quality control (QC) was based on four control genes (*CHMP2A* (charged multivesicular body protein 2A), *GPI* (glucose-6-phosphate isomerase), *RAB7A* (RAB7A, member RAS oncogene family), and *VCP* (valosin containing protein)) as indicators of RNA quality and content. Gene fusion variants were further evaluated using Alamut Visual Plus (v1.12; SOPHiA GENETICS, Inc., Boston, MA, USA). Single-nucleotide variants were identified based on total read depth ≥ 100, ≥10 alternate observations, and a variant allelic frequency ≥ 5%. Gene fusions were called if supported by ≥5 unique reads, ≥10% total reads, and ≥3 unique start sites [80].

All variants were assessed for their impact on the coding region (missense, nonsense, frameshift) and clinical significance (pathogenic, likely pathogenic, benign, likely benign, uncertain) using Catalogue of Somatic Mutations in Cancer (COSMIC), v103 [81], ClinVar [82] and VarSome, v13.13.1 [83]. Therapeutic evidence was determined via OncoKB, v6.0 [84,85], the Cancer Knowledgebase (CKB; Genomenon, Ann Arbor, MI, USA), and the My Cancer Genome database [86]. The origin (somatic or germline) of the SNVs was evaluated according to previously reported data (COSMIC, ClinVar, and VarSome) and the allele frequencies estimated in Non-Finnish European populations in the Genome Aggregation Database (gnomAD), v.2.1.1 [87], using a cut-off of ≥4.0 × 10^−4^ for germline variants [88].

Synonymous variants were excluded from the final analysis. Variants were annotated according to the Human Genome Variation Society (HGVS) nomenclature [89] and are described in the text using HGVS protein (HGVSp) or HGVSp short-form notation. The distribution of variants was visualized using the OncoPrinter tool in cBioPortal (v6.3.1) [90,91]. Structural modeling of the fusion protein was based on the ORF reconstruction from RNA fusion junction, reference transcript comparison (RefSeq), InterPro [92] and Pfam protein domain databases [93], and AlphaFold Protein Structure Database [94,95].

### 4.7. Statistical Analysis

Descriptive statistics, including the median and range (min–max), were used to summarize the technical performance of the NGS assay. Clinicopathological characteristics and the frequency of genetic variants in LCNEC patients were compared using the Pearson’s chi-squared test and the Kruskal–Wallis H test. All analyses were performed using MedCalc Statistical Software (v23.4.5; Ostend, Belgium). Statistical significance was defined as *p* < 0.05.

## 5. Conclusions

Our study results revealed that up to 13% of patients with LCNEC could potentially benefit from personalized treatment strategies involving either approved or investigational targeted therapies. The most common genetic alterations were found in the *KRAS* and *PIK3CA* genes, less common in the *RET*, *EGFR*, *BRAF*, and *NTRK1* genes. Despite the current lack of standardized treatment and molecular testing protocols for pulmonary LCNEC, our data align with existing literature suggesting that a significant portion of these patients are candidates for targeted therapy. Future research should focus on establishing an optimal gene panel for LCNEC, alongside prospective clinical trials to validate the efficacy of personalized medicine in this aggressive histological subtype of lung carcinoma.

## Figures and Tables

**Table 1:** The summary of unique single nucleotide variants detected in pulmonary large-cell neuroendocrine carcinoma (LCNEC) tissue samples and possible targeted treatment options based on approved or experimental drugs.

Gene	HGVSc	HGVSp	Variant Position GRCh37 (hg19)	dbSNP	Variation ID (ClinVar)	Allele Frequency (GnomAD)	Molecular Consequence (ClinVar)	Pathogenicity (ClinVar/VarSome)	Oncogenicity (OncoKB/CKB)	Biological Effect (OncoKB)	FDA/EMA- Approved Drug in NSCLC	Experimental Drug in NSCLC
*BRAF*	c.1406G>C	p.Gly469Ala	chr7:140481402	rs121913355	13971	0.000	Missense	Pathogenic	Oncogenic	Gain-of-function		Plixorafenib
*BRAF*	c.1743T>A	p.Asn581Lys	chr7:140453192	rs397516895	44811	n/a	Missense	Pathogenic/Likely pathogenic	Likely Oncogenic	Unknown		
*EGFR*	c.988G>A	p.Glu330Lys	chr7:55223621	rs139429793	560008	0.00003553	Missense	Conflicting classifications	Oncogenic	Gain-of-function		Nimotuzumab
*EGFR*	c.1792G>A	p.Gly598Arg	chr7:55233042	rs2128946294	2582257	n/a	Missense	Pathogenic	Likely Oncogenic	Unknown		
*EGFR*	c.2227G>T	p.Ala743Ser	chr7:55242457	rs759256622		n/a	Missense	Pathogenic Supporting (in silico)	Unknown	Unknown		
*KRAS*	c.34G>T	p.Gly12Cys	chr12:25398285	rs121913530	12578	0.000	Missense	Likely Pathogenic	Oncogenic	Gain-of-function	Adagrasib, Sotorasib	
*KRAS*	c.35G>T	p.Gly12Val	chr12:25398284	rs121913529	12583	0.000	Missense	Pathogenic	Oncogenic	Gain-of-function		Daraxonrasib, Binimetinib, Cobimetinib, Trametinib
*KRAS*	c.34_35delins CT	p.Gly12Leu	chr12:25398284	rs2135806231		n/a	DELINS		Likely Oncogenic	Likely Gain-of-function		Binimetinib, Cobimetinib, Trametinib
*KRAS*	c.27_35delins AGGACCTGT	p.Ala11_Gly12 delinsProVal	chr12:25398284			n/a	DELINS		Unknown	Unknown		Daraxonrasib Binimetinib, Cobimetinib, Trametinib
*MET*	c.1124A>G	p.Asn375Ser	chr7:116340262	rs33917957	41611	0.01847	Missense	Benign	Inconclusive	Inconclusive		
*MET*	c.2908C>T	p.Arg970Cys	chr7:116411923	rs34589476	41623	0.005042	Missense	Conflicting classifications	Inconclusive	Inconclusive		
*PIK3CA*	c.1624G>A	p.Glu542Lys	chr3:178936082	rs121913273	31944	n/a	Missense	Pathogenic	Oncogenic	Gain-of-function		Alpelisib, RLY-2608
*PIK3CA*	c.1633G>A	p.Glu545Lys	chr3:178936091	rs104886003	13655	0.000008879	Missense	Likely Pathogenic	Oncogenic	Gain-of-function		Alpelisib, RLY-2608
*PIK3CA*	c.1636C>A	p.Gln546Lys	chr3:178936094	rs121913286	13657	n/a	Missense	Likely Pathogenic	Oncogenic	Gain-of-function		Alpelisib, RLY-2608
*PIK3CA*	c.3140A>G	p.His1047Arg	chr3:178952085	rs121913279	13652	0.000008910	Missense	Pathogenic	Oncogenic	Gain-of-function		Alpelisib, RLY-2608
*PIK3CA*	c.3140A>T	p.His1047Leu	chr3:178952085	rs121913279	13653	0.000008910	Missense	Pathogenic	Oncogenic	Gain-of-function		Alpelisib, RLY-2608
*RET*	c.1825T>G	p.Cys609Gly	chr10:43609069	rs77558292	3254419	n/a	Missense	Pathogenic	Likely Oncogenic	Likely Gain-of-function		Selpercatinib, Pralsetinib
*RET*	c.1946C>T	p.Ser649Leu	chr10:43609994	rs148935214	24928	0.0005887	Missense	Conflicting classifications	Likely Oncogenic	Likely Gain-of-function		Selpercatinib, Pralsetinib
*RET*	c.2071G>A	p.Gly691Ser	chr10:43610119	rs1799939	24934	0.1847	Missense	Benign	Inconclusive	Unknown		Dovitinib (TKI258)
*RET*	c.2372A>T	p.Tyr791Phe	chr10:43613908	rs77724903	13936	0.002048	Missense	Likely Benign	Inconclusive	Unknown		
*RET*	c.2753T>C	p.Met918Thr	chr10:43617416	rs74799832	13919	0.000008791	Missense	Pathogenic	Oncogenic	Gain-of-function		Selpercatinib, Pralsetinib

*BRAF*—B-raf proto-oncogene, serine/threonine kinase; *EGFR*—epidermal growth factor receptor kinase; *KRAS*—KRAS proto-oncogene, GTPase; *MET*—MET proto-oncogene, receptor tyrosine kinase; *PIK3CA*—phosphatidylinositol-4,5-bisphosphate 3-kinase catalytic subunit alpha; *RET*—rearranged during transfection proto-oncogene; n/a = data not available. Reference transcripts: NM_004333.6 for *BRAF*, NM_005228.5 for *EGFR*, NM_004985.5 for *KRAS*, NM_000245.4 for *MET*, NM_006218.4 for *PIK3CA*, NM_020975.6 for *RET* gene. Reference genome version: GRCh37 (hg19).

**Table 2:** The clinicopathological characteristics of LCNEC patients from whom tumor samples were obtained for molecular analysis.

Characteristics		N (%)
Patients with lung carcinoma		216 (100%)
Median age (range), years	70 (47–90)	
Sex		
Female		100 (46%)
Male		116 (54%)
Histology (WHO)		
LCNEC		216 (100%)
Stage (cTNM)		
operable (I-IIIA)		66 (31%)
advanced (IIIB-IV)		150 (69%)

## Data Availability

The detailed data presented in this study are available from the corresponding authors on reasonable request.

## Supplementary Materials

The following supporting information can be downloaded at: https://www.mdpi.com/article/10.3390/ijms27072939/s1.
