# Supplementary material for: Identification of Actionable Gene Variants in Pulmonary Large-Cell Neuroendocrine Carcinoma: A Real-World Analysis of a Polish Cohort
Source: Int J Mol Sci. 2026 Mar 24;27(7):2939. doi: 10.3390/ijms27072939 (PMC13073004; doi:10.3390/ijms27072939)
Supplement: Supplementary file 1 [file ijms-27-02939-s001.zip › ijms-4173610-supplementary.pdf]

SUPPLEMENTARY MATERIAL

Table S1. The summary of gene variants detected by FusionPlex Lung v1 Panel (Integrated Sciences, Chatswood, CA, USA).

Gene	Accession	Covered Exons	Variant Type	Detected Variants (HGVSp)
ALK	NM_004304	N/A	Mutation	p.T1151-p.C1156, p.F1174, p.L1196-p.S1206, p.G1269
ALK	NM_004304	2, 4, 6, 10, 16, 17, 18,19,20, 21, 22, 23, 26	Fusion	5'
BRAF	NM_004333	N/A	Mutation	p.V600
BRAF	NM_004333	2, 7, 8, 9, 10, 11, 12, 15, 16	Fusion	5'
BRAF	NM_004333	1, 3, 7, 8, 10, 13	Fusion	3′
EGFR	NM_005228	N/A	Mutation	p.E709-p.G719, p.E746-p.L760, p.V774-p.G796, p.L858-p.L861
EGFR	NM_005228	7, 8, 9, 16, 19, 20,	Fusion	5'
EGFR	NM_005228	8	Exon 2-7 Skipping (EGFRvIII)	5′
EGFR	NM_005228	1, 24, 25	Fusion	3'
EGFR	NM_005228	1	Exon 2-7 Skipping (EGFRvIII)	3'
FGFR1	NM_015850	2, 3, 4, 5, 6, 7, 8, 9, 10, 11, 17	Fusion	5′
FGFR1	NM_015850	12, 17	Fusion	3'
FGFR2	NM_000141	2, 5, 7, 8, 9, 10	Fusion	5'
FGFR2	NM_000141	16, 17	Fusion	3'
FGFR3	NM_000142	3, 5, 8, 9, 10	Fusion	5′
FGFR3	NM_000142	16, 17, 18	Fusion	3′
KRAS	NM_004985	2, 3	Mutation	p.G12-p.G13, p.Q61
MET	NM_000245	2, 4, 5, 6, 13, 14, 15, 16, 17,21	Fusion	5′
MET	NM_000245	15	Exon 14 Skipping	5′
MET	NM_000245	2, 13	Fusion	3′
MET	NM_000245	13	Exon 14 Skipping	3′
NRG1	NM_013957	1, 8	Fusion	5′
NRG1	NM_004495	1, 2, 3, 4, 6	Fusion	5′
NRG1	NM 013962	1	Fusion	3′
NTRK1	NM_002529	2, 4, 6, 8, 10, 11, 13	Fusion	5′
NTRK2	NM_006180	5, 7, 9, 11, 12, 13, 15, 16, 17	Fusion	5′
NTRK3	NM_002530	4, 7, 10, 12, 13, 14, 15, 16	Fusion	5′
NTRK3	NM 001007156	15	Fusion	5′
NTRK3	NM_002530	13, 14, 15	Fusion	3′
RET	NM_020630	N/A	Mutation	p.A883, p.M918
RET	NM_020975	8, 9, 10, 11, 12, 14	Fusion	5′
RET	NM_020630	2, 4, 6	Fusion	5′
ROS1	NM 020630	N/A	Mutation	p.G2032
ROS1	NM_002944	2, 4, 7, 31, 32, 33,	Fusion	5′

Table S2. The summary of gene variants detected by FusionPlex Lung v2 Panel (Integrated Sciences, Chatswood, CA, USA).

Gene	Accession	Covered Exons	Variant Type	Detected Variants (HGVSp)
ALK	NM_004304	2, 4, 6, 8, 10, 12, 14, 16, 17, 18, 19, intron19, 20, mid-exon20, 21, 22, 23, 26	Fusion, Internal deletion (ALKΔ2-17, ALKΔ2-3)	5'
ALK	NM_004304	1, 2	Internal deletion (ALKΔ2-17, ALKΔ2-3)	3'
ALK	NM_004304	22, 23, 25	Mutation	p.P1153-p.C1156, p.F1174, p.L1196-p.S1206, p.G1269
BRAF	NM_004333	2, 3, 4, 5, 7, 8, 9, 10, 11, 12, 15, 16	Fusion, Kinase Domain Duplication, BRAFΔ2-10, BRAFΔ4-10,BRAFΔ2-8, BRAFΔ3-8,BRAFΔ4-8	5'
BRAF	NM_004333	1, 2, 3, 7, 8, 10, 13, 14, 18	Fusion, Kinase Domain Duplication, BRAFΔ2-10, BRAFΔ4-10,BRAFΔ2-8, BRAFΔ3-8,BRAFΔ4-8	3'
BRAF	NM_004333	15	Mutation	p.V600
EGFR	NM_005228	7, 8, 9, 14, 15, 16, 17, 18, 19, 20	Fusion, Exon 2-7 Skipping (EGFRvIII), Kinase Domain Duplication	5'
EGFR	NM_005228	1, 24, 25, mid-exon 25, 26	Fusion, Exon 2-7 Skipping (EGFRvIII), Kinase Domain Duplication	3'
EGFR	NM_005228	18, 19, 20, 21	Mutation	p.E709-p.G719, p.E746-p.L760, p.V774-p.G796, p.L858-p.L861
ERBB2	NM_004448	4, 5, 13, 15, 17	Fusion, Exon 16 skipping (Δ16HER)	5'
ERBB2	NM_004448	15, 23, 24, 25, mid-exon 26, 26	Fusion, Exon 16 skipping (Δ16HER)	3'
ERBB2	NM_004448	8, 20	Mutation	p.G309-p.S310, p.Y772-p.P780, p.C805
FGFR1	NM_015850	2*, 3, 4, 5, 6, 7, 8, 9, 10, 11, 17	Fusion, Kinase Domain Duplication	5'
FGFR1	NM_015850	12, 17	Fusion, Kinase Domain Duplication	3'
FGFR1	NM_023110	4, 13, 14	Mutation	p.T141, p.V561, p.K656
FGFR2	NM_000141	2*, 3, 5, 6, 7, 8, 9, 10	Fusion	5'
FGFR2	NM_000141	16, 17, 18	Fusion	3'
FGFR2	NM_000141	7, 9, 12, 13, 14	Mutation	p.S252-p.P253, p.G305, p.Y375-V395, p.I547-p.N549, p.V564, p.A648-p.K659
FGFR3	NM_000142	3, 5, 8, 9, 10, 11, 12, 13, 14	Fusion	5'
FGFR3	NM_000142	16, 17, intron 17, mid-exon 18	Fusion	3'
FGFR3	NM_000142	7, 9, 13, 14, 16	Mutation	p.R248-p.S249, p.G370-p.R399, p.V555, p.D641-p.K650, p.G697-p.K715
KRAS	NM_004985	2, 3, 4	Mutation	p.G12-p.G13, p.Q61, p.K117, p.A146
MET	NM_000245	2, 4, 5, 6, 13, 14, 15, 16, 17, 21	Fusion, Exon 14 Skipping (METΔex14)	5'
MET	NM_000245	2, 13	Fusion, Exon 14 Skipping (METΔex14)	3'
NRG1	NM_001159996	1*, 3, 4, 5	Fusion	5'
NRG1	NM_004495	1, 2, 3, 4, 5, 6	Fusion	5'
NRG1	NM_013958	1*	Fusion	5'

NRG1	NM_013959	1*, 3	Fusion	5'
NRG1	NM_013962	1*	Fusion	5'
NRG1	NM_013962	1	Fusion	3'
NTRK1	NM_001007792	1, 2	Fusion	5'
NTRK1	NM_002529	1, 2, 3, 4, 5, 6, 7, 8, 9, 10, 11, 12, 13, 14	Fusion	5'
NTRK1	NM_002529	13, 14, 15, 16, 17	Mutation	Full kinase domain coverage for resistance mutations including p.G595
NTRK2	NM_006180	3, 5, 6, 7, 8, 9, 10, 11, 12, 13, 14, 15, 16, 17, 18	Fusion	5'
NTRK2	NM_006180	11, 14	Fusion	3'
NTRK2	NM_006180	16, 17, 18, 19, 20, 21	Mutation	Full kinase domain coverage for resistance mutations
NTRK3	NM_001007156	15	Fusion	5'
NTRK3	NM_002530	3, 4, 5, 6, 7, 8, 9, 10, 11, 12, 13, 14, 15, 16	Fusion	5'
NTRK3	NM_002530	13, 14, 15, 17	Fusion	3'
NTRK3	NM_002530	15, 16, 17, 18, 19	Mutation	Full kinase domain coverage for resistance mutation detection including p.F617, p.G623, p.G696
NUTM1	NM_175741	2*, 3, mid-exon 3, 4, 5, mid-exon 6, 6	Fusion	5'
PIK3CA	NM_006218	2, 15	Fusion	5'
PIK3CA	NM_006218	2, 3, 5, 6, 8, 10, 14, 21	Mutation	p.E81K-p.G118D, p.L339-p.D350, p.G364R, p.E418-p.C420, p.E453-p.K468, p.P539-p.Q546, p.E726, p.Y1021-p.T1052
RET	NM_020630	2, 4, 6, 8, 9, 10, 11, mid-exon 11, 12, 13, 14	Fusion	5'
RET	NM_020630	15, 16	Mutation	p.A883, p.M918
ROS1	NM_002944	2, 4, 7, 31, 32, 33, 34, 35, 36, 37	Fusion	5'
ROS1	NM_002944	38	Mutation	p.G2032

*Indicates exons that are entirely untranslated region (UTR), or for which the UTR is targeted.

Table S3. The summary of single nucleotide and fusion variants identified in pulmonary large-cell neuroendocrine carcinomas using targeted next generation sequencing.

Patient No.	Sex (M/F)	Age (years)	Specimen type	Tumor cell content in FFPET	Stage (cTNM)	Gene	Variant on cDNA (HGVSc)	Variant on protein (HGVSp)	Variant Allele Frequency	Coverage (total reads)
#1.	M	81	CNB	70%	IV	*BRAF*	c.1406G>C	p.Gly469Ala	25%	1100
#2.	F	73	Resection	85%	IA3	*BRAF*	c.1743T>A	p.Asn581Lys	6%	492
#3.	M	77	Resection	80%	IIIA	*EGFR*	c.988G>A	p.Glu330Lys	47%	102
#4.	F	69	VATS biopsy	20%	IIIA	*EGFR*	c.1792G>A	p.Gly598Arg	5%	489
#5.	M	56	VATS biopsy	50%	IIIB	*EGFR*	c.2227G>T	p.Ala743Ser	59%	637
#6.	F	72	CNB	90%	II	*KRAS*	c.34G>T	p.Gly12Cys	46%	5653
#7.	F	64	Bronchoscopy biopsy	90%	IVA	*KRAS*	c.34G>T	p.Gly12Cys	15%	1754
#8.	F	72	EBUS TBNA	90%	IIIA	*KRAS*	c.34G>T	p.Gly12Cys	6%	350
#9.	M	70	CNB	80%	IIIB	*KRAS*	c.34G>T	p.Gly12Cys	54%	2192
#10.	M	67	Resection	70%	IB	*KRAS*	c.34G>T	p.Gly12Cys	64%	929
#11.	M	78	CNB	40%	II	*KRAS*	c.34G>T	p.Gly12Cys	57%	6056
#12.	M	61	TBNA	60%	IIIC	*KRAS*	c.35G>T	p.Gly12Val	5%	491
#13.	M	65	FNA	70%	IV	*KRAS*	c.35G>T	p.Gly12Val	5%	478
#14.	M	69	Bronchoscopy biopsy	10%	IIIB	*KRAS*	c.35G>T	p.Gly12Val	68%	5872
#15.	F	74	CNB	50%	IVB	*KRAS*	c.34_35delins CT	p.Gly12Leu	34%	9845
#16.	M	79	CNB	60%	IIIA	*KRAS*	c.27_35delins AGGACCTGT	p.Ala11_Gly12 delinsProVal	46%	7560
#17.	F	66	Intraoperative biopsy	40%	IVA	*MET*	c.1124A>G	p.Asn375Ser	15%	104
#18.	M	62	CNB	80%	IV	*MET*	c.1124A>G	p.Asn375Ser	28%	346
#19.	F	75	FNA	100%	IV	*MET*	c.1124A>G	p.Asn375Ser	48%	8667
#20.	F	69	Resection	90%	II	*MET*	c.1124A>G	p.Asn375Ser	51%	2436
#21.	F	70	USG FNA	90%	IIIB	*MET*	c.1124A>G	p.Asn375Ser	33%	238
#22.	F	81	CNB	90%	I	*MET*	c.1124A>G	p.Asn375Ser	50%	1260
#23.	M	53	EBUS FNA	40%	IVB	*MET*	c.1124A>G	p.Asn375Ser	39%	368
#24.	F	64	EBUS FNA	90%	IIIC	*MET*	c.1124A>G	p.Asn375Ser	96%	599
#25.	M	68	EBUS FNA	60%	IV	*MET*	c.1124A>G	p.Asn375Ser	31%	988

#26.	M	74	CNB	5%	IA	*MET*	c.1124A>G	p.Asn375Ser	39%	265
#27.	F	67	Bronchoscopy biopsy	80%	IV	*MET*	c.1124A>G	p.Asn375Ser	57%	1616
#28.	M	67	CNB	50%	IV	*MET*	c.1124A>G	p.Asn375Ser	50%	1205
#29.	M	71	EBUS FNA	90%	IIIB	*MET*	c.1124A>G	p.Asn375Ser	49%	963
#30.	M	68	Bronchoscopy biopsy	60%	IV	*MET*	c.1124A>G	p.Asn375Ser	52%	982
#31.	M	59	Bronchoscopy biopsy	60%	IV	*MET*	c.2908C>T	p.Arg970Cys	67%	155
#32.	M	68	TBNA	70%	IIIA	*PIK3CA*	c.1624G>A	p.Glu542Lys	21%	105
#33.	M	73	CNB	80%	I	*PIK3CA*	c.1633G>A	p.Glu545Lys	94%	201
#34.	F	76	Resection	40%	IIIA	*PIK3CA*	c.1633G>A	p.Glu545Lys	39%	100
#35.	M	71	Resection	80%	IIB	*PIK3CA*	c.1633G>A	p.Glu545Lys	8%	266
#36.	M	72	CNB	80%	IIIA	*PIK3CA*	c.1636C>A	p.Gln546Lys	33%	243
#37.	M	81	Resection	50%	IIB	*PIK3CA*	c.3140A>T	p.His1047Leu	20%	1405
#38.	F	75	Bronchoscopy biopsy	80%	IV	*PIK3CA*	c.3140A>G	p.His1047Arg	26%	737
#39.	M	69	CNB	70%	I	*PIK3CA*	c.3140A>G	p.His1047Arg	26%	1359
#40.	F	85	CNB	80%	IV	*RET*	c.1825T>G	p.Cys609Gly	45%	658
#41.	F	65	Bronchial brush smear	90%	IIIC	*RET*	c.1946C>T	p.Ser649Leu	53%	272
#42.	M	73	Resection	30%	IIIA	*RET*	c.1946C>T	p.Ser649Leu	56%	1207
#43.	F	74	Resection	90%	IIB	*RET*	c.2071G>A	p.Gly691Ser	98%	1775
#44.	F	63	CNB	80%	IVB	*RET*	c.2372A>T	p.Tyr791Phe	45%	2382
#45.	F	68	Bronchoscopy biopsy	80%	II	*RET*	c.2753T>C	p.Met918Thr	100%	2534
#46	F	72	USG FNA	80%	IVB	Fusion *TMEM79::NTRK1*	t(1;1), NM_032323.3 exon A: 3; NM_002529.3 exon B: 2; (chr1:156256264,chr1:156834146)		66%	

CNB – Core Needle Biopsy; VATS – Video-Assisted Thoracoscopic Surgery; TBNA - transbronchial needle aspiration; EBUS-TBNA – Endobronchial Ultrasound-guided Transbronchial Needle Aspiration; FNA – Fine Needle Aspiration; USG-FNA – Ultrasound-guided Fine Needle Aspiration; EBUS-FNA – Endobronchial Ultrasound-guided Fine-Needle Aspiration; *BRAF* – B-raf proto-oncogene, serine/threonine kinase; *EGFR* – epidermal growth factor receptor kinase; *KRAS* – KRAS proto-oncogene, GTPase; *MET* – MET proto-oncogene, receptor tyrosine kinase; *PIK3CA* – phosphatidylinositol-4,5-bisphosphate 3-kinase catalytic subunit alpha; *RET* - rearranged during transfection proto-oncogene; *TMEM79* – transmembrane protein 79; NTRK1 – neurotrophic receptor tyrosine kinase 1.
